# Worms’ Antimicrobial Peptides

**DOI:** 10.3390/md17090512

**Published:** 2019-08-29

**Authors:** Renato Bruno, Marc Maresca, Stéphane Canaan, Jean-François Cavalier, Kamel Mabrouk, Céline Boidin-Wichlacz, Hamza Olleik, Daniela Zeppilli, Priscille Brodin, François Massol, Didier Jollivet, Sascha Jung, Aurélie Tasiemski

**Affiliations:** 1 Univ. Lille, CNRS, Inserm, CHU Lille, Institut Pasteur de Lille, U1019 – UMR 8204 - CIIL - Center for Infection and Immunity of Lille, F-59000 Lille, France; 2 Univ. Lille, CNRS, UMR 8198 - Evo-Eco-Paleo, F-59000 Lille, France; 3 Aix-Marseille Univ, CNRS, Centrale Marseille, iSm2, F-13013 Marseille, France; 4 Aix-Marseille Univ, CNRS, LISM, IMM FR3479, F-13009 Marseille, France; 5 Aix-Marseille Univ, CNRS, UMR7273, ICR, F-13013 Marseille, France; 6 IFREMER Centre Brest REM/EEP/LEP, ZI de la Pointe du Diable, CS10070, F-29280 Plouzané, France; 7 Sorbonne Université, CNRS, UMR 7144 AD2M, Station Biologique de Roscoff, Place Georges Teissier CS90074, F-29688 Roscoff, France; 8 Department of Applied and Molecular Microbiology, Institute of Biotechnology, Technische Universität Berlin, 13355 Berlin, Germany

**Keywords:** Antibiotics, annelids, nematodes, AMP, extremophiles

## Abstract

Antimicrobial peptides (AMPs) are natural antibiotics produced by all living organisms. In metazoans, they act as host defense factors by eliminating microbial pathogens. But they also help to select the colonizing bacterial symbionts while coping with specific environmental challenges. Although many AMPs share common structural characteristics, for example having an overall size between 10–100 amino acids, a net positive charge, a γ-core motif, or a high content of cysteines, they greatly differ in coding sequences as a consequence of multiple parallel evolution in the face of pathogens. The majority of AMPs is specific of certain taxa or even typifying species. This is especially the case of annelids (ringed worms). Even in regions with extreme environmental conditions (polar, hydrothermal, abyssal, polluted, etc.), worms have colonized all habitats on Earth and dominated in biomass most of them while co-occurring with a large number and variety of bacteria. This review surveys the different structures and functions of AMPs that have been so far encountered in annelids and nematodes. It highlights the wide diversity of AMP primary structures and their originality that presumably mimics the highly diverse life styles and ecology of worms. From the unique system that represents marine annelids, we have studied the effect of abiotic pressures on the selection of AMPs and demonstrated the promising sources of antibiotics that they could constitute.

## 1. Introduction

Antimicrobial peptides (AMPs) are natural antibiotics produced by all living organisms, from archaea to mammals [1,2,3]. In pluricellular organisms, they act as key actors of immunity by operating in the first line of defense towards microbes [4,5,6,7] such as bacteria, fungi, and protozoa or viruses that attempt to invade and to proliferate into the host [8,9,10,11,12]. AMPs also contribute to symbiostasis (i.e., the regulation of mutualistic and commensal symbionts to avoid proliferation) in vertebrates and invertebrates by controlling, shaping, and confining the symbiotic microflora in specific anatomical compartments (gut, bacteriomes, skin) [13,14,15]. Because symbionts have been shown to represent a rapid source of innovation for the host to adapt to changing habitats, AMPs are also indirectly involved in the ability of animals and plants to cope with environmental changes [16,17,18,19]. In metazoans, active AMPs are generally matured from a larger inactive protein precursor containing a signal peptide, a proregion, and the AMP itself. The ribosomal synthesis and/or the secretion of AMPs by epithelial and circulating cells are well documented to be regulated by microbial challenges, while few data also evidence an influence of abiotic factors. However, there are increasing examples of an endogenous role of AMPs, i.e., they are active towards the expressing host and work as cannibal toxins [20].

Regarding their application as type of therapeutic drugs, after their first discovery in the early 1980s, AMPs appeared as a promise of novel antibiotics to address issues about the multi-drug resistance (MDR) of pathogenic bacteria. Animals are the most important producers of AMPs (2298 versus 349 from plants or 342 from bacteria), although very poorly described in worms, with only 20 AMPs discovered out of seven species [21]. The definition of an AMP is only based on physico-chemical criteria (<100 amino acids in length, amphipathic, cationic) and on their properties to kill microbes. Recently, a unifying structural signature present in cysteine-stabilized AMPs was discovered: The γ-core motif [22]. Virtually all peptides sharing the γ-core motif interact with the negatively-charged lipid membranes causing ion-channel dysfunction or membrane pore formation in bacteria. One important point is that the multi-target interaction and mechanism of action (MOA) of AMPs with the bacterial membrane makes the appearance of resistance to AMPs more difficult compared to conventional antibiotics. AMPs and AMP-resistance mechanisms have presumably co-evolved through a transitory host–pathogen balance that has characterized the existing AMP collection [23]. Additional bioactivity features of AMPs such as their natural antibacterial biofilm activities, their chemotaxis of immune cells, immunomodulation, endotoxin neutralization, their mediation of nerve-repair activities [23] also add value/benefit to AMPs compared to conventional antibiotics [3,11,24]. However, most of the existing sequences of AMPs have never been exploited so far. Thirty years after their discovery, a better understanding of their MOA, modifications (structural and/or residues substitution), and synthesis is reigniting the commercial development of AMPs, which “stage a comeback” [25].

The production of AMPs and their contribution to host immunity have been well demonstrated in worms (Table 1) [2,12,26,27,28,29,30]. Their involvement in resistance to microbial infection and in symbiostasis is sustained by their strategic location in immune cells (phagocytes), in body fluids (pseudo-coelom, coelom, and blood) and at the interfaces between organisms and their environment, *i.e.*, at epithelial cell levels such as intestinal cells and epidermis cells. The first worm AMP (namely cecropin P1) was isolated and identified in 1989 [31] by the team of H. Boman, who just discovered the existence of AMPs in the butterfly *Hyalophora cecropia* [32]. The cecropin P1 was originally thought to be a porcine cecropin until the workers who isolated it provided evidence in 2003 that, in fact, this AMP originated from the pig intestinal parasitic nematode *Ascaris suum*, and not from its mammalian host [19]. This underlines the non-negligible difficulty and importance of separating host DNA, RNA, or peptides from those of potential parasites and symbionts when searching for a new component. To date, cecropins have been identified mainly in ecdysozoans (insects and nematodes), in one marine tunicate, and in bacteria [18,33,34], but neither in lophotrochozoans (molluscs, annelids, etc.) nor in vertebrates.

In 1996, ABF-type peptides (also called nematode defensins) were discovered in nematodes by Kato et al. [35]. Like mollusc and insect defensins, they contain eight cysteine residues and harbor a cysteine-stabilized alpha helix and beta sheet (CSαβ) structure. These common features may suggest an evolution from a common ancestor [36]. However, the lack of a significant sequence similarity or a conserved genomic organization (exon–intron structure) suggests that these groups of AMPs have rather emerged through convergent evolution [37]. In 1998, Banyai and Patthy demonstrated the antibacterial activities of saposin-like proteins (SPP) (called caenopores) from *Caenorhabditis elegans*, a family of AMPs similar to the amoebapores of the unicellular *Entamoeba histolytica* and the granulysin from human cytotoxic T lymphocytes [38]. Amoebapore-like SPPs might have been the first AMPs since this family emerged in protists, i.e., before the advent of multi-celled organisms [39]. In 2002, Mallo et al. observed, in *C. elegans* again, the induced expression of a neuropeptide-like peptide (nlp) upon bacterial infection. Later, in 2004, Ewbank’s group indirectly demonstrated an antifungal activity for nlp-31 [40]. Until now, nlps have not been identified in non-nematode species, and their MOA and 3D structures remain to be solved. As detailed below, other AMPs were identified in nematodes, but, to our knowledge, except for cecropins, none of these were purified from crude extracts of worms; their predicted “in silico” sequences are issued from homology-based searches in genomes or transcriptomes starting from already described AMP sequences in other invertebrates [41]. Due to the rapid molecular evolution and high diversity of AMPs, one can assume that not all families of AMPs are characterized yet in nematodes. Efforts were also mainly focused on *C. elegans* and should be extended to wild species and enlarged to different taxa of nematodes. However, one major problem when searching for new AMPs from nematodes, as we have tried with the marine *Metoncholaimus* and *Oncholaimus* spp., is their tiny size (0.2 mm diameter) combined with their highly variable and patchy distribution in their natural habitat, making it complicated and not reproducible the collect of a sufficient number of individuals. Although promising at first, the too low quantity of material was a clear limitation to the use of the bioassay-guided purification, which remains the best and only strategy to discover new AMPs (unpublished data).

By contrast to nematodes, most annelid AMPs were biochemically isolated from diverse wild species from different taxa. The first annelid AMP was lumbricin-1 isolated from the earthworm *Lumbricus rubellus* in 1998 [42] and later in leeches [43]. Its MOA, as well as its 3D structure, have yet to be described. The relatively low antimicrobial activities of lumbricin-like AMPs suggest that the microbial clearance is not the main biological function of this molecule. In 2004, the first member of the macin family (theromacin) was characterized in leeches [44]. Despite their different disulfide arrays, macins and invertebrate defensins share the CSαβ motif also characteristic of the members of the scorpion toxin-like superfamily [6]. By contrast with defensins, macins have been shown to exert neurotrophic and proliferation effects, in addition to their bactericidal activities [6,43]. Based on their functions, their expression sites, their occurrence, and their evolutionary relationship in the animal kingdom, the possibility to consider macins as defensins could be discussed. Another family of cysteine-rich AMPs was characterized in annelids: The BRICHOS (so called from Bri2, CHOndromodulin, and proSurfactant protein C) AMP family; the first member was arenicin isolated from the body fluid of *Arenicola marina* in 2004 [14]. At this time, the presence of a BRICHOS domain in the proregion of the arenicin precursor was not noticed by the authors and was first mentioned later in 2013 in a review written by Knight et al. who discovered the BRICHOS domain in 2002 [45,46]. The evidence of other members and the study of their gene evolution confirm the existence of the BRICHOS-AMP family, which seems to be restricted to marine worms [47]. Even if AMPs from this family do not share any sequence similarity, they harbor a beta hairpin structure stabilized by one or two disulphide bridges [48].

This review surveys the wide diversity of primary and tertiary structures of worm-produced AMPs as a consequence of a hundred millions years of worms’ evolution and diversification and natural selection occurring at the interspecific level according to peculiar lifestyles and habitats. We focus on annelids, which represent the worm clade for which the research of AMPs has not been targeted on genetic/laboratory models as performed in nematodes, but is rather the result of species exploration over a variety of environments (marine, terrestrial, freshwater, etc.). This review highlights that none of the AMP families are universally expressed and that none of the studied worm species seem to produce all types of AMPs, even if the lack of genomes does not allow to firmly confirm this observation. Thus, the exploration and study of novel and unconventional worm species appear as a promising source of new AMPs and of different modes of immune defense in link with the ecology/habitat of the species of interest.

## 2. AMPs Diversity in Annelids and Nematodes

AMPs’ capacity to kill microorganisms lies in their ability to disrupt and/or permeate the target cell membranes. Being generally cationic, they usually accumulate at the membrane surface (negatively charged) of the bacteria. Then, above a certain concentration threshold, they disrupt the cell membrane through very diverse and complex mechanisms [9]. Most of the MOAs studied act via pore formation (barrel-stave or toroidal models) or by non-pore mechanisms, such as a carpet-like mechanism. In both the pore models, at increasing concentrations, peptides begin to orientate perpendicular to the membrane and insert into the bilayer: In the toroidal model, the peptides are always associated with the lipid head groups; in the barrel-stave model, they form a bundle in the membrane with a central lumen (the peptides represent the staves of the barrel) [9,52].

Alternatively, in the carpet model, the peptides cover the membrane surface in a carpet-like manner (orientated in parallel to the membrane) and at high concentrations, they disrupt the bilayer in a detergent-like manner, leading to the formation of micelles [53]. Some AMPs polarize the membrane, forming anionic lipid clusters [54]. A minority of AMPs, however, do not cause membrane disruption: After crossing the bacterial cell membrane, they act on intracellular targets (such as nucleic acids and functional proteins) to activate cell death [55].

AMPs can be classified into several subgroups according to their secondary structure and biochemical characteristics: (i) α-helix peptides, containing one or more helices with spatially disjunct hydrophobic and hydrophilic surfaces [56]; (ii) β-sheet peptides, with β-hairpin-like structure, rich in cysteine and containing disulfide bonds; (iii) α-helix/β-sheets peptides with mixed α-helical and β-sheet organization [4,57]; (iv) extended peptides, which do not adopt regular secondary structures, containing a high proportion of one or two amino acids (such as proline, glycine, tryptophan, etc.) often essential for their antimicrobial activity [57,58]; and (v) peptides derived from larger molecules, exerting multiple functions [59]. Interestingly, representatives from all of these structural groups have been identified in worms (summarized in Table 2). They represent the main subject of this article and are subsequently described below.

### 2.1. α-helix Peptides

#### 2.1.1. α-helix Peptides in Nematodes

##### Cecropin and Caenopore Families

**Figure 1:**
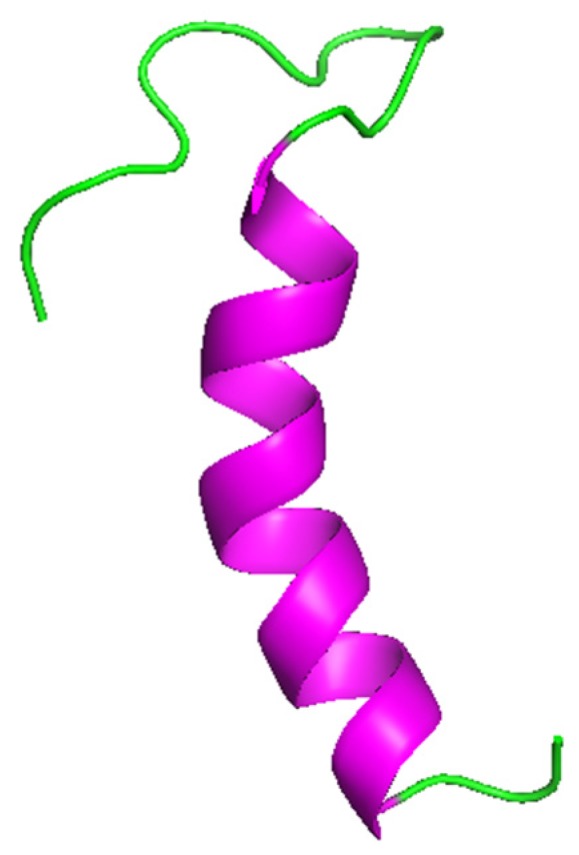
Three-dimensional structure of cecropin-P1, representative of cecropin family (PDB ID: 2N92) [31]. Picture generated using PyMOL (TM) 2.3.2 software: BioLuminate, Schrödinger, LLC, New York, NY, USA 2019 (www.pymol.org).

Cecropins and cecropin-like peptides have been identified and characterized in insects [60,61], nematodes [19,29], tunicates [18], and bacteria [34]. In worms, cecropins have only been detected in the nematode *Ascaris suum* (cecropin-P1, -P2, -P3 and -P4), a pig intestinal parasite, and other species of *Ascarididae* (at least in *A. lumbricoides* and *Toxocara canis*) [19,62]. These AMPs are short in length, rich in serine, not stabilized by disulfide bonds, and display a linear and amphipathic α-helical structure (Figure 1) [29,63]. 

**Figure 2:**
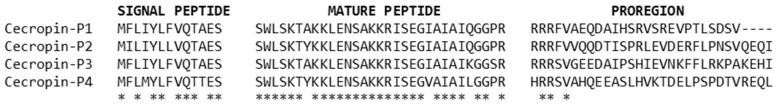
Sequence alignment of cecropin-family from *A. suum*; * conserved amino acids.

Cecropins are derived from precursor molecules, with a common structure, i.e., having a signal peptide, a mature peptide, and a pro-region (Figure 2) [64]. As for α-defensins (mammalian AMPs), the acidic pro-region may inhibit the antimicrobial/cytotoxic activity of the basic mature region, protecting the cells of AMP production sites [65]. The primary structures of the mature cecropins are highly conserved and consist of 31 residues [62]. 

*Ascaris* cecropins exhibit potent antimicrobial activity. They are upregulated upon bacterial challenge and are active against Gram-positive bacteria (*Staphylococcus aureus*, *Bacillus subtilis*, *Micrococcus luteus*), Gram-negative bacteria (*Pseudomonas aeruginosa*, *Salmonella typhimurium*, *Escherichia coli*), and also fungi (*Saccharomyces cervisae*, *Candida albicans)* (Table 3) [62,66,67]. 

The interaction between cecropin and the bacterial membrane is initiated by the C-terminal α-helical structure that plays a crucial role in lipopolysaccharide recognition. Cecropins exert pore formation as a bacterial-killing mechanism [33].Recently, disease-resistant fish and shellfish strains were produced by transgenesis of cecropins-P1 gene, exhibiting elevated resistance to infection by different pathogens [68,69]; cecropin-P4 was used against chicken and pig pathogens as a food supplement to livestock production [70]. 

**Figure 3:**
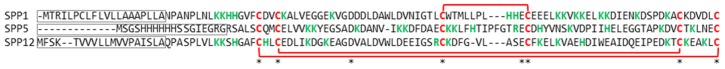
Sequence alignment of caenopores (or saposins); signal peptide in the frame; in red bold type, cysteine residues involved in disulfide bonds; in green bold type, cationic residue; * conserved amino acids.

Caenopores (from *Caenorhabditis elegans*) belong to the saposin-like protein (SAPLIP) superfamily, a group of small proteins of different sizes and various cellular functions [71]. They are cationic peptides, characterized by the conserved positions of six cysteine residues involved in the formation of three disulfide bonds (Figure 3) [29]. Twenty-three different caenopore-coding genes have been evidenced in *C. elegans*, but antimicrobial activities have only been described for caenopore-1 (SPP-1), caenopore-5 (SPP-5), and caenopore-12 (SPP-12) [72,73,74].

**Figure 4:**
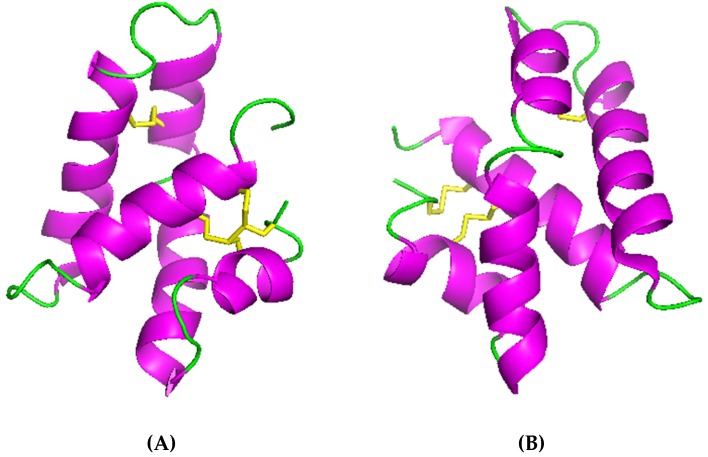
Three-dimensional structure of the SSP-5 conformers: (**A**) *Cis* isomer (PDB ID: 2JS9) [77]; (**B**) *Trans* isomer (PDB ID: 2JSA) [77]. Helices in purple and disulfide bridges in yellow. Pictures generated using PyMOL (TM) 2.3.2 software (www.pymol.org).

These three molecules are active against *Bacillus megaterium*; moreover SPP-5 shows significant activity against *E. coli* and SPP-12 is active against *B. thuringiensis* (Table 3) [72,75]. As reported by several authors, natural variants in this AMP family (33 AMPs encoded by 28 different genes) are inducible by different microbes and have a different target spectrum against bacteria and fungi [72,73,76]. Under acidic conditions (pH 5.2), these AMPs are able to form pores, leading to the permeabilization of the bacterial membranes [72]. SSP-5 and SSP-1 are exclusively expressed in the intestine, probably to kill ingested bacteria, and SPP-12 is exclusively expressed in the two pharyngeal neurons [73,75]. In general, it seems that they contribute to both the digestion and the immune defense of the host [73]. To date, only the 3D structure of SSP-5 has been solved at 0.6 Å of resolution, revealing the existence of two conformers (Figure 4). 

**Figure 5:**
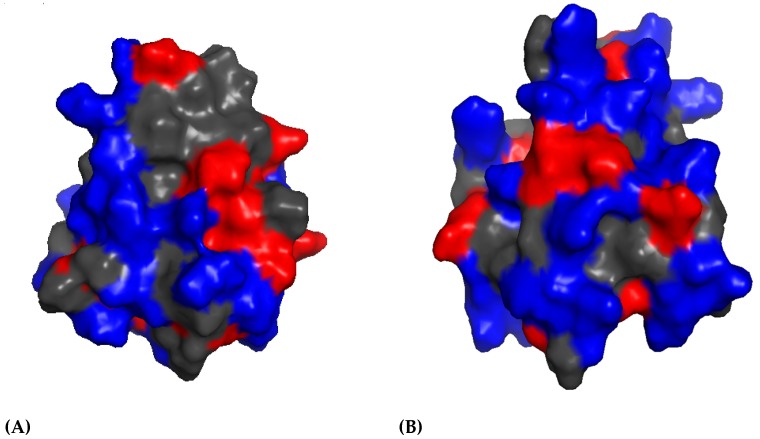
SSP-5 *cis* (**A**) and *trans* (**B**) 3D structures of the surface. Hydrophobic, charged, and polar residues are represented in grey, blue, and red, respectively. Pictures generated using PyMOL (TM) 2.3.2 software (www.pymol.org).

The *cis* and *trans* conformers (differing in the isomerization of the peptide bond between Cys80 and Pro81) consist of a bundle of five amphipathic helices which are arranged in a folded leaf with two halves [77]. The 3D structures of both conformers display a large hydrophobic region and an uniformly distributed charged residue covering the surface (Figure 5). SSP-5 was found to exert its antibacterial activity by pore formation (as already shown for amoebapore-like peptides which also belong to the SAPLIP family) [77].

#### 2.1.2. α-helix Peptides in Annellids

##### Hedistin

Hedistin is a linear peptide, identified from the marine annelid *Hediste diversicolor* [50]. To date, no hedistin-like sequences have been found in other species. This ragworm is an euryhaline marine polychaete (order of *Phyllodocida*) able to withstand great variations in salinity. Hedistin (primary structure: LGAW_Br_LAGKVAGTVATYAW_Br_NRYV) is the only annelid peptide containing bromotryptophan residues. As shown for cathelicidin peptides, this modification might be the result of an adaptation that makes the AMP less vulnerable to proteolysis for steric reasons [50,78]. It also carries a C-terminal amidation that increases the cationic charge, and thus its attraction for negatively charged bacterial membranes [50,79]. Hedistin is active against Gram-positive bacteria (especially *Micrococcus luteus* and *Micrococcus nishinomiyaensis*) and the Gram-negative bacterium *Vibrio alginolyticus* (Table 3) [50]. The 3D structure presents three segments, forming a helix–bend–helix conformation that suggests bacterial membrane disruption through a carpet model [50,80]. Hedistin is constitutively and strongly produced by NK-like cells circulating in the body cavity of annelids [50]. 

### 2.2. β-sheet Peptides in Annelids

#### BRICHOS-AMPs Family

**Figure 6:**
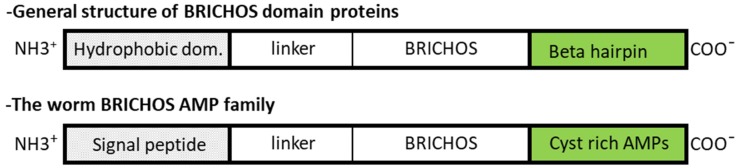
Structural organization of the precursor of a BRICHOS-AMP.

Surprisingly, members of this AMP family have been identified in polychaetes only. These AMPs are processed from a larger precursor containing a BRICHOS domain (Figure 6) [14,48,81]. This domain consists of 100 amino acids and the different BRICHOS family members always show the following structure (Figure 6): (i) A hydrophobic region (a signal peptide or a transmembrane region), (ii) a proregion with a linker and a BRICHOS domain, and (iii) a C-terminal region whose amino-acid residues fold into a double stranded β-sheet (a cysteine rich AMP). While present in a wide range of organisms, the functional properties of the BRICHOS domain has only been explored in mammals [71]. 

In humans, BRICHOS is a constituent of protein families associated with amyloid formation, found in several major human diseases (Alzheimer’s, Parkinson’s, diabetes mellitus, dementia, respiratory distress, and cancer) [48,82]. The BRICHOS family member proSP-C (prosurfactant protein C), although the most studied, has no antimicrobial activity due to the absence of the C-terminal extension, i.e., the AMP part. However, in case of proSP-C, BRICHOS binds to the amyloidogenic transmembrane region, preventing it from self-aggregating. The second well studied protein, Bri2, possesses the general structure of BRICHOS family proteins. Current data show that the Bri2 domain interacts as a molecular chaperone on its C-terminal extension (Bri23) to maintain a β-hairpin structure, which has no antimicrobial activity either [82].

In marine annelids, by contrast with the relatively well conserved BRICHOS domain, the AMP part of the precursor shows a high diversity with sequences that do not share any homologies, suggesting that a strong selection at the interspecific level has probably occurred probably in link with the habitat of the worms [47]. The first discovered members of this family were arenicin-1 and arenicin-2 [14], isolated from the coelomocytes of *Arenicola marina*, a coastal polychaete. This lugworm inhabits sand flats, characterized by high fluctuations of temperature, salinity, oxygen, and sulphide concentrations [83]. The primary structures of the two cyclic isoforms differ only by one amino acid substitution (Val10Ile). They are characterized by 21 residues with a single disulfide bond that connects the N- and C-terminus (Cys3 – Cys20). Later, a third isoform, termed arenicin-3, showing significant differences in the sequence from the first two arenicins and containing one additional disulfide bond (Cys7 – Cys16) was isolated and characterized [84]. Another member of this AMP family named alvinellacin was isolated later and identified from *Alvinella pompejana* the emblematic Pompeii worm that inhabits the hottest part of the black chimneys of the deep eastern Pacific ocean [81]. This animal is considered as the most thermotolerant and eurythermal animal in the world, facing bursts of elevated temperatures as high as 80 °C but also harsh acidic conditions and high pressures (up to 300 bars) [85]. In such a fluctuating and extreme environment, genetic analysis of alvinellacin has given evidence of an adaptive diversification of the molecular chaperone of the AMP, but not of the AMP itself, as the result of the gain of a vital and highly conserved epsilon proteobacteria ectosymbiosis in the face of the joint thermal and sulfide fluctuations of the vent habitat [47]. Biochemical characterization of alvinellacin has revealed that its primary structure is composed of 22 amino acid residues and stabilized by two disulfide bonds [48,86]. However, it is worth noting that BRICHOS-AMP homologs have been also described in other alvinellid and terebellid worms that do not always exhibit bacterial epibioses, and thus represent a very ‘old’ family of AMPs in annelids.

**Figure 7:**
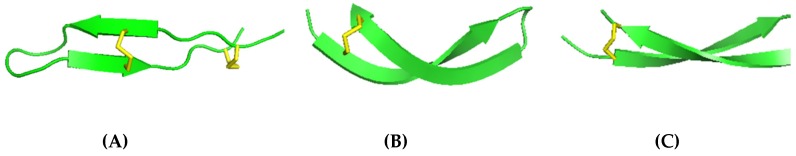
Three-dimensional structure of (**A**) alvinellacin (PDB ID: 2LLR) [48], (**B**) arenicin-1 (PDB ID: 2JSB) [89], and (**C**) arenicin-2 (PDB ID: 2JNI) [88]. Disulfide bridges in yellow. Pictures generated using PyMOL (TM) 2.3.2 software (www.pymol.org).

As mentioned above, annelid AMPs with BRICHOS are characterized by a short amino-acid sequence, a cationic net charge, a hydrophobic region, a β-sheet fold, and the formation of disulfide bonds between cysteine residues, increasing the rigidity of their open-ended cyclic structures (Table 4) [87,88,89]. Different specific software can easily determine all these structural characteristics. The Innovagen Pepcalc.com server (Innovagen AB, SE-22370 Lund, SWEDEN; https://pepcalc.com/) was used to calculate the net charge at neutral pH, and Peptide2.0 server (Peptide 2.0 Inc., Chantilly, VA; https://peptide2.com/) to evaluate the peptide hydrophobicity. The positive charge (due to arginine residues) and the hydrophobicity (from valine, leucine, alanine, tryptophan, isoleucine, phenylalanine, and tyrosine) contribute to the amphipathic nature of the peptide. In aqueous solution, they adopt a β-hairpin conformation, formed by two twisted antiparallel β-strands, stabilized by intra-backbone hydrogen bonds and one or two disulfide bonds between cysteine residues (Figure 7) [48,88,89,90]. This motif was found in other AMPs, like protegrins, gomesin, and tachyplesins, but not in combination with a large residue ring structure (showed in Figure 7) [91,92,93].

Notably, the structural properties of BRICHOS-AMPs are linked to their membranolytic activity, exhibiting a broad spectrum of activities against Gram-positive, Gram-negative bacterial, and fungal pathogens (Table 3) [94]. Arenicin isoforms display potent antibacterial activity against Gram-positive bacteria (*Listeria monocytogenes*, *Staphylococcus aureus, Staphylococcus epidermidis, Planococcus citreus, Bacillus subtilis, Bacillus megaterium, Micrococcus luteus*), Gram-negative bacteria (*E. coli, Klebsiella pneumoniae, Salmonella enterica, Salmonella typhimurium, Pseudomonas aeruginosa, Proteus mirabilis*, *Vibrio alginolyticus*, *Listonella anguillarum, Agrobacterium tumefaciens*), and also antifungal activity (*Candida albicans, Fusarium solani*) [14,28,88,89,90,95,96,97,98,99,100]. Alvinellacin is active against Gram-positive bacteria (*B. megaterium* and *S. aureus*) and Gram-negative bacteria (*E. coli*, *V. diabolicus*, *Pseudomonas* sp., *V. MPV19*). Interestingly, in contrast to the majority of known AMPs, the antimicrobial activity of arenicin-family members is preserved in the presence of salt [14,48,89]. Similarly, low temperature conditions (+4 °C) do not impede arenicin-1 antimicrobial inhibition on *E. coli* and *P. mirabilis* [89].

The peptides kill a number of bacterial strains within minutes by membrane permeabilization, membrane detachment, and release of cytoplasm [14,89]. The mechanism of action of arenicins is still under investigation, and recent studies propose a “toroidal-pore” model, including monomeric or dimeric peptide organization [98,101,102]. The AMP interaction with the anionic phospholipidic bilayer of bacterial membranes is promoted by the high abundance of hydrophobic and positively-charged residues [98,102,103]. The binding to the membranes leads to conformational changes of the peptide molecule [28,104]. Two N-terminal β-strands of peptides associate to form a dimer mediating pore formation [28,101,104]. In yeast, arenicin-1 may act indirectly, inducing apoptosis via intracellular accumulation of reactive oxygen species, and directly damages mitochondria and DNA in nuclei [105].

Except for alvinellacin, which is not hemolytic or cytotoxic to mammalian cells, arenicins are cytotoxic to human cell lines and cause hemolysis of human red blood cells. Although this precludes their development as candidate antimicrobials, artificial modified analogs were designed based on their structure, in order to decrease their adverse effects and to enhance the antimicrobial properties. Novel derivatives named NZ17074, N2, and N6 were designed and synthesized as linear or with more disulfide bonds by amino acid substitution [90,97,106,107]. By showing a higher antimicrobial activity and a lower cytotoxicity, these latter derivatives were more powerful than the parent molecule. Therefore, these positive results suggest these AMPs as potential candidates for antibacterial drug development [81,107,108].

Arenicin-1 and 2 and alvinellacin transcripts are expressed constitutively in coelomocytes, in the body wall, the foregut, and midgut, suggesting a peptide’s involvement in both systemic and epithelial branches of immunity [14,83,109]. These AMPs are also present in a major part of the nervous system, which suggests a possible involvement in the defense and the regeneration of the nerve cord as demonstrated for the cysteine rich AMPs of the leeches (see below) [43,89,109]. Data given also evidences that alvinellacin shapes and controls the specific epibiotic microflora that allows it to thrive in the hydrothermal habitat [48].

Recently, nicomicin-1 and -2 were identified in the artic polychaeta *Nicomache minor* [110]. This worm lives in the cold water, inhabiting hard tubes attached to stones [111]. Nicomicins consist of 33 residues (Table 1), containing BRICHOS domain in the sequences of their prepropeptide. They are characterized by many hydrophobic amino acids (51%) and a disulfide bond (Cys24 – Cys29) [110]. While Nicomicin-2 has no effect on bacteria, Nicomicin-1 exerts strong antimicrobial activity towards Gram-positive bacteria by damaging their membranes; the presence of salt impedes its activity [110]. Conversely, the AMP 3D structure is different from alvinellacin and arenicin and is organized into two independent regions with an α-helix at the N-terminal moiety and a six-residue loop stabilized by the disulfide bridge at the C-terminus [110].

### 2.3. Mixed α-helix/β-sheet Peptides

#### 2.3.1. Mixed α-helix/β-sheet Peptides in Nematodes

##### The ABF Family

**Figure 8:**
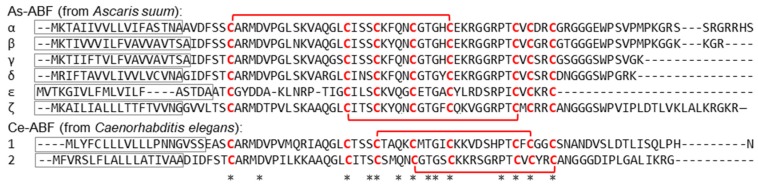
ABF family members’ sequence alignment: Signal peptide in the frame; in red bold type, cysteine residues involved in disulfide bridges; * conserved amino acids.

ABFs (antibacterial factors) are defensin-like AMPs characterized in nematodes only, first in *Ascaris suum* (seven As-ABFs) and then in *Caenorhabditis elegans* (five Ce-ABFs), in *Ancylostoma duodenale* (six Ad-ABFs), and one Cbr-ABF in *C. briggsae* [35,112,113]. This family of peptides appears to be widely distributed in nematodes (86 peptides from 25 species) with different lifestyles and habitats. *A. suum* and *A. duodenale* are hematophageous parasitic, living in the small intestine of mammalian hosts; *C. elegans* and *C. briggsae* are not parasitic and inhabit compost and garden soil. Despite their similarities with macins, they have not been found in annelids. Nematode defensins are cationic and cysteine rich peptides, with formation of disulfide bonds (Figure 8) [114,115,116].

**Figure 9:**
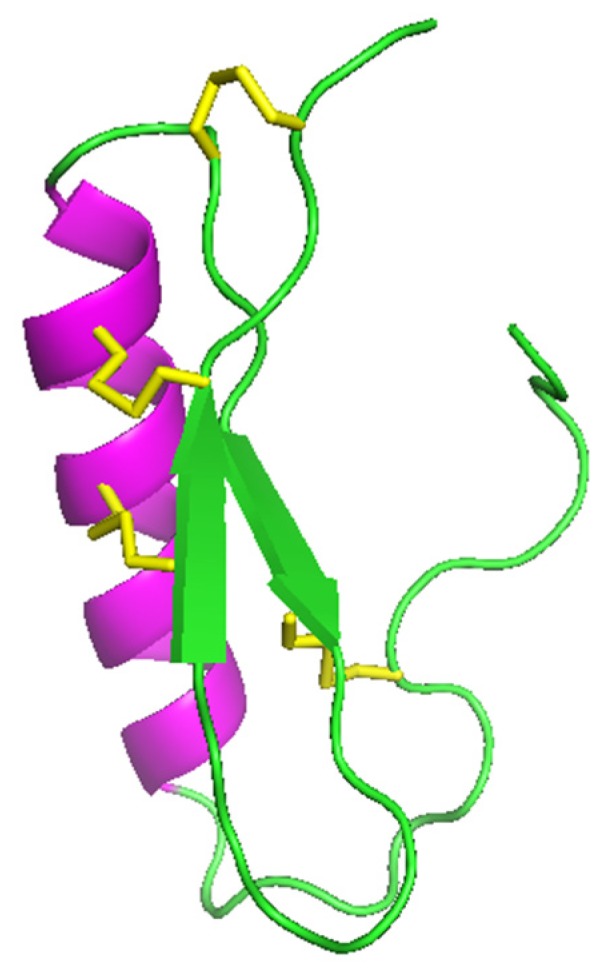
Three-dimensional structure of the As-ABF-alpha (PDB ID: 2D56): In green, antiparallel β-sheets; in purple, α-helix; and in yellow, disulfide bridges [35]. Picture generated using PyMOL (TM) 2.3.2 software (www.pymol.org).

Although the structure for As-ABF-α is the only one having been experimentally determined (Figure 9), the ABFs’ structural motif is characterized by an α-helix and two β-sheets stabilized by three disulfide bonds (CS-αβ), the fourth bond contributes to the firmness of the open ended cyclic molecule [4,64].

The antibacterial activity has been screened for As-ABF-alpha and Ce-ABF2 only, and both exhibit higher antimicrobial activity against Gram-positive bacteria (through pore formation) than against Gram-negative bacteria and yeast (Table 3); the presence of salt inhibits their bactericidal activity [35,112,113,114,117]. Their expression increases upon bacterial challenge [73,116]. As-ABFs have been detected mainly in the body wall and in other tissues, probably with diversified physiological roles [116]. Conversely, Ce-ABF1 and Ce-ABF2 are mainly produced in the pharynx of *C. elegans*, i.e., the site where live bacteria accumulate after their ingestion [113].

#### 2.3.2. Mixed α-helix/β-sheet Peptides in Annelids

##### Macin Family

**Figure 10:**
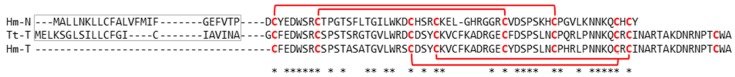
Sequence alignment of Macin family members. Signal peptide in the frame; in red bold type, cysteine residues involved in disulfide bonds; * conserved amino acids.

Macins are cationic cysteine-rich AMPs. Members of this family of peptides have been first described in leeches (*Theromyzon tessulatum* and *Hirudo medicinalis*) [43,44], and later in *Hydra vulgaris* [43,118] and in the mollusks *Hyriopsis cumingii* [80] and *Mytilus galloprovincialis* [119]. Both leeches belong to the “Clitellata” class: *T. tessulatum* is a shallow water rhynchobdellid leech, ectoparasite of aquatic birds [120]; *H. medicinalis*, a gnathobdellid leech, is an ectoparasite of mammals which lives in stagnant freshwater and streams [121]. Tt-theromacin (Tt-T) in *T. tessulatum* [44], Hm-neuromacin (Hm-N) and Hm-theromacin (Hm-T) in *H. medicinalis* [43], have several functions that includes bacterial killing, symbiostasis in the gut, immune defense, and regeneration of the damaged nerve cord. Their primary structure is highly conserved with the presence of a signal peptide (except for Hm-Theromacin), four disulfide bridges [122], and a fifth intramolecular disulfide bond (C31:C73) in theromacins (Figure 10) [118]. 

**Figure 11:**
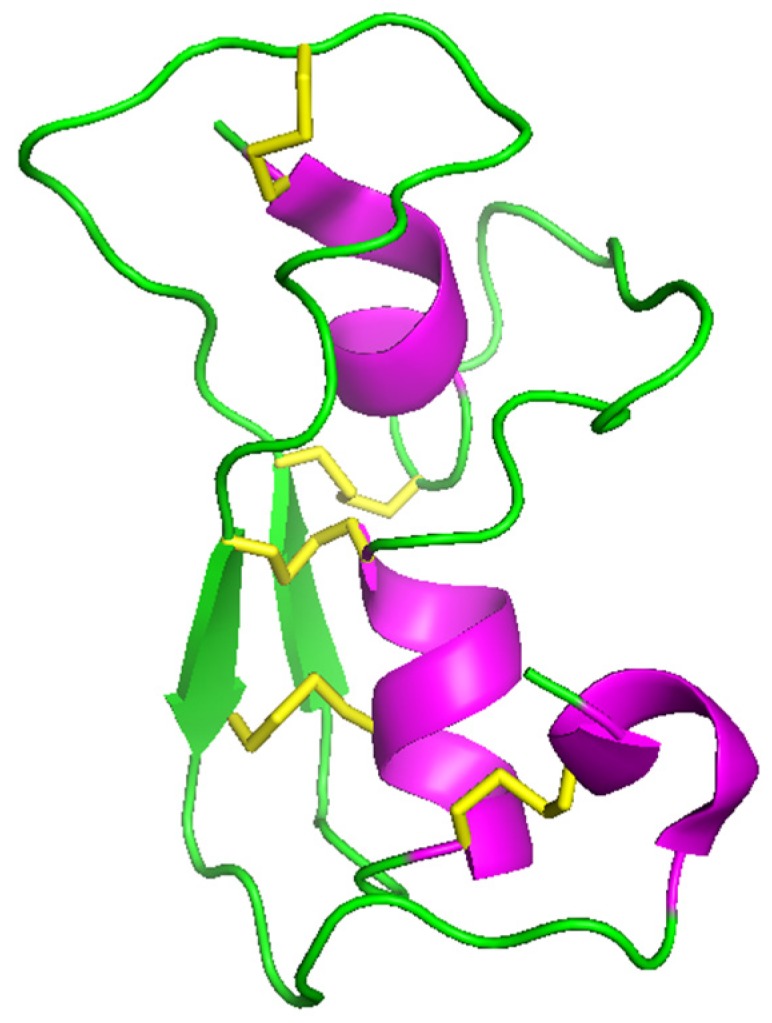
Three-dimensional structure of Tt-theromacin (PDB ID: 2LN8): In green, antiparallel β-sheets; in purple, α-helix; and in yellow, disulfide bridges [6]. Picture generated using PyMOL (TM) 2.3.2 software (www.pymol.org).

Macin peptides represent rather long and complex peptides of more than 60 residues. The tertiary structure of macin family members is organized in a knottin-fold according to the arrangement of cysteine bonds, and the peptides’ molecular surfaces are divided into two hydrophobic hemispheres (due to the band-like distribution of the positive charges) [118,122]. Figure 11 shows the open-ended cyclic structure of theromacin. The conserved structural features in the macin family are an additional α-helix in N-terminal position and two long flexible loops, distinguishing them from all other peptides of the scorpion-toxin like superfamily in which the macin family belongs [118]. 

Theromacin and neuromacin have been evidenced to display antimicrobial activity against Gram-positive bacteria (*B. megaterium* and *M. luteus*) [6] and low antibacterial activity against Gram-negative proteobacteria (*E. coli*) [44]; neuromacin being also active against *Micrococcus nishinomiyaensis* (Table 3) [43]. The activities are impeded with increasing salt concentrations [6]. The MOA of the family (barnacle model) includes the permeabilization of the membrane of Gram-positive bacteria, but also the pore formation as observed for neuromacin [6]. Thanks to their structural double-amphipathic character (two hydrophobic hemispheres sandwiched by a belt of positive charges), initially macins promote aggregation of bacteria, and after, they permeabilize the bacterial membrane [6,118].

In addition to antibacterial activity, both neuromacin and theromacin exert nerve-cord regeneration activity [6,43]. In *H. medicinalis*, theromacin is released in the blood surrounding the nervous system, and neuromacin is produced by nerve cells and accumulates at the wounded site of the central nervous system [123], whereas Tt-theromacin is expressed in large fat cells and released immediately into the coelomic fluid following infections or damages of the central nervous system [6,44].

### 2.4. Peptides Enriched with One or Two Specific Amino Acids

#### 2.4.1. Peptides Enriched with One or Two Specific Amino Acids in Nematodes

##### Neuropeptide-Like Peptides and Caenacins

**Figure 12:**
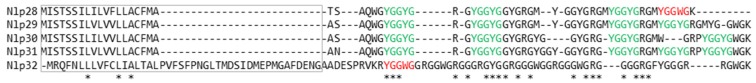
Nlp family members’ sequence alignment: Signal peptide in the frame; YGGYG and YGGWG motifs in green and red, respectively; * conserved amino acids.

**Figure 13:**
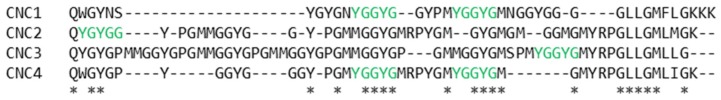
Caenacin members’ sequence alignment: YGGYG motifs in green; * conserved amino acids.

Neuropeptide-like peptides (nlps) and caenacins (CNCs) are basic peptides which are enriched in glycine and aromatic amino acids residues [40,124,125]. They are induced in the hypodermis by infection (i.e., *Drechmeria coniospora*) or wounding in *C. elegans* and other nematodes species, playing diverse roles in nervous system functioning [125]. These two AMP groups represent 111 genes already known. In Figure 12 and Figure 13, some examples of nlp and CNC families are listed, showing YGGWG and YGGYG motifs which are likely to typify this group of AMPs [126].

Interestingly, the expression of nlp and CNC genes in *C. elegans* is downregulated upon challenge with the majority of tested bacteria and upregulated in the case of fungal infections [73]. To date, there have been no direct tests of the antimicrobial/antifungal activity for these peptides and the MOA has not yet been described. Recently, nlp-31 exhibited activity towards *Burkholderia pseudomallei* (a Gram-negative bacterium, resistant to a wide range of antimicrobials), thereby binding to DNA and interfering with bacterial viability without any membrane disruption activity [127].

#### 2.4.2. Peptides enriched with one or two specific amino acids in annelids 

##### Lumbricin Family

Lumbricins are proline-rich AMPs characterized in oligochaete earthworms *Lumbricus rubellus* (lumbricin-1), *Pheretima tschiliensis* (PP-1), *P. guillelmi* (lumbricin-PG), and *Eiseinia andrei* (Lumbr and LuRP), and in the leech *H. medicinalis* (Hm-lumbricin) [42,43,128,129,130], but also found in polychaetes such as the Pompeii worm *A. pompejana* (AT & DJ, pers. obs.) and leeches [88]. 

**Figure 14:**
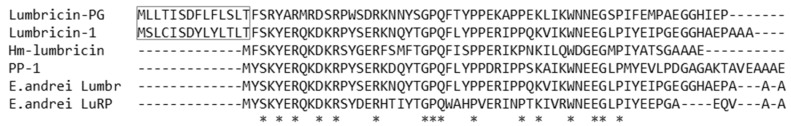
Lumbricin family members’ sequence alignment: Signal peptide in the frame; * conserved amino acids.

Their amino acid sequences include numerous prolines and aromatic amino-acid residues (phenylalanine, tyrosine or tryptophan); only lumbricin-1 and lumbricin-PG exhibit a signal peptide sequence (Figure 14). 

Lumbricin-1 exhibits antibacterial activity towards a broad spectrum of Gram-positive and Gram-negative bacteria and fungi (Table 3) [42,43,128,129,130], but their MOA is still unknown. PP-1 is synthesized in the mucus of the epidermis; the two lumbricins from *E. andrei* have been detected in the intestine and in other tissues (body wall, gut, ovary, etc.) [43,128,131,132]. Interestingly, Hm-lumbricin gene expression is rapidly enhanced by bacterial challenge [43], whereas Lumbr and LuRP are slowly induced (after 48 h) following the infection [129]. By contrast, lumbricin-1 (present only in adult worms) is not inducible when the animal is subjected to a bacterial challenge [42]. Hm-lumbricin exerts neuroregenerative properties in leeches, as observed for neuromacin [43]. Nowadays, the tertiary structures of lumbricins, nlps, and CNCs have not been solved [114].

### 2.5. Peptides Derived from Larger Molecules in Annelids

#### 2.5.1. Perinerin

Perinerin is a cationic, hydrophobic, and linear peptide, isolated and characterized from the Asian marine clamworm *Perinereis aibuhitensis* (Grube, 1878) [49,133]. This annelid is a marine polychaete, living in the sediment of estuaries [134]. Perinerin consists of 51 amino-acid residues (primary structure: FNKLKQGSSKRTCAKCFRKIMPSVHELDERRRGANRWAAGFRKCVSSICRY), with a high proportion of arginine and four cysteine residues possibly involved in the formation of two disulfide bonds [49]. Despite the presence of cysteine residues and disulfide bonds, the Perinerin sequence does not show any similarities with the previously described AMPs in annelids, and its average sequence identity to other cysteine-rich AMPs is less than 30% [135]. It exhibits a broad range of antimicrobial activities (antifungal, bactericidal against Gram-negative and Gram-positive bacteria) without any observed microbial resistance (Table 3) [49]. The proposed MOA is pore-forming activity and the bactericidal action against the Gram-positive bacteria *B. megaterium* is very fast (less than 3 minutes) [79]. Perinerin purification is obtained from unchallenged individuals, and suggests that the peptide is constitutively expressed [49]. Until now, no studies describing the three-dimensional structure of Perinerin have been performed.

#### 2.5.2. Ms-Hemerycin

Ms-Hemerycin is an AMP from the polychaete *Marphysa sanguinea*, a marine lugworm that inhabits mudflats [51]. Its amino-acid sequence consists of 14 amino acids (Ac-SVEIPKPFKWNDSF) blocked by a N-terminal acetylation for its stability. Ms-Hemerycin is derived from the split of the N-terminus of the well-known respiratory pigment hemerythrin found in several marine invertebrates. This peptide exhibits potent activity against Gram-negative and Gram-positive bacteria (Table 3). Ms-Hemerycin has been detected constitutively in all examined tissues, with higher concentration in brain and muscle. The secondary structure might be unordered, containing a partial α-helical region. From such an unordered structure, it can be predicted that the MOA should be very different from the other AMPs [30,51].

## 3. Conclusions and Perspectives

Among biological models, marine worms are particularly attractive for searching and studying the adaptation/evolution of AMPs to environmental conditions despite their high level of divergence. Compared to the terrestrial environment, the sea has remained virtually unexplored for its ability to yield pharmacological metabolites. In the last decades, research has expanded from lands to oceans in order to find new drug candidates. Because the oceans occupy almost 70% of Earth’s surface, they offer a vast potential for biological and chemical diversities. Even more interesting are marine worms living in extreme habitats. The peculiar thermochemical and biotic pressures (and notably, the abundance of Gram-negative bacteria where most actual MDR bacteria belong to) that marine worms have to face in hostile environments represent a natural laboratory to select AMPs able to be more acid-resistant, thermostable, salt-tolerant, and active against most bacterial strains. Extremophile worms constitute interesting models to search and study novel drugs [136]. 

Moreover, the study of AMPs produced by extremophile annelids offers the perspective to add an initial piece in the complex relationship between the external immunity of the host and its ectosymbionts recruitment and growth control [48,137,138].

## Figures and Tables

**Table 1:** Dates of antimicrobial peptides’ (AMPs) discovery in nematodes and in annelids.

Worm Phylum	Dates	AMP Families	References
Nematodes	1989	Cecropins	[31]
	1996	ABFs	[35]
	1998	Coenopores	[38]
	2002	Caenacins	[39]
	2004	Nlps	[40]
Annelids	1998	Lumbricins	[42]
	2004	Macins	[44]
	2004 and 2013	BRICHOS-AMPs	[14,46]
	2004	Perinerin	[49]
	2006	Hedistin	[50]
	2016	Ms-Hemerycin	[51]

**Table 2:** Repartition of the different groups of identified AMPs according to the phylum and the respective habitats of the worms.

Structure (Group)	AMPs	Worm Phylum	Worm Habitat
Linear α-helix (i)	Cecropins	Nematode	Terrestrial
	Caenopores	Nematode	Terrestrial
	Hedistin	Annelid	Marine
β-sheet (ii)	BRICHOS-AMPs	Annelid	Marine
Mixed α-helix/β-sheet (iii)	ABFs	Nematode	Terrestrial
	Macins	Annelid	Freshwater
Enriched with specific amino acids (iv)	Neuropeptide-like	Nematode	Terrestrial
	Caenacins	Nematode	Terrestrial
	Lumbricins	Annelid	Marine and Freshwater
Derived from larger molecules (v)	Perinerin	Annelid	Marine
	Ms-Hemerycin	Annelid	Marine

**Table 3:** Antimicrobial activity spectrum of worm AMPs. The values are expressed in µM: MIC (Minimal Inhibitory Concentration) in black, MBC. (Minimal Bactericidal Concentration) in red, and B.C.50 (50% Bactericidal Concentration) in green.

Microorganisms		CECROPINS				CAENOPORES			HEDISTIN	BRICHOS FAMILY				ABFS		MACINS			LUMBRICINS		PERINERIN
		P1	P2	P3	P4	SSP1	SSP5	SSP12		arenicin1	arenicin2	alvinellacin	nicomicin1	AS-α	CE-2	Hm-N	Tt-T	Hm-T	PG	1	
*G* *R* *A* *M* *N* *E* *G* *A* *T* *I* *V* *E*	*Escherichia coli*	0.3–0.5	30	9	20		0.1		0.8–1.6	4	4	0.012–0.024	2–16	50		25	25		20	12	12.5–25
	*Pseudomonas aeruginosa*	0.4–0.5	20	20	20					2			32								3.1–9.2
	*Pseudomonas sp.*											0.001–0.003									
	*Salmonella enterica*									0.6											
	*Salmonella typhimurium*	0.4–0.5	20	8	8																
	*Proteus mirabilis*									0.6											
	*Proteus vulgaris*													10							
	*Klebsiella pneumoniae*	0.5								2–4				70	0.9						
	*Vibrio alginolyticus*									0.4											
	*Vibrio diabolicus*											0.048–0.096									
	*Vibrio MPV19*											0.012–0.024									
	*Listonella anguillarum*									3.1											
	*Bdellovibrio bacteriovorus*													0.5	0.06						
	*Agrobacterium tumefaciens*										5			10	0.05						
	*Serratia sp.*																		2.5	16	
*G* *R* *A* *M* *P* *O* *S* *I* *T* *I* *V* *E*	*Micrococcus luteus*	8	30	8	8				0.4–0.8		2.6		0.125	0.8			0.165–0.33				25–50
	*Micrococcus nishinomiyaensis*								0.4–0.8									1.95–3.8			
	*Staphylococcus aureus*	22.2	8	3	3				3–6	2–8		0.048–0.096	2	0.6		6.25	100		5	16	
	*Staphylococcus epidermidis*									4–8											
	*Streptococcus mutans*																			30	
	*Bacillus megaterium*					0.1	0.05	0.275			2.6	0.012–-0.024				0.20	0.39				2.5-5
	*Bacillus subtilis*	2	20	10	20					0.31			0.062	1.2						12	
	*Bacillus thuringiensis*							10													
	*Kocuria varians*													0.5	0.008						
	*Enterococcus faecium*	3.4–4								12.5											
	*Enterococcus faecalis*	9.4																			
	*Planococcus citreus*									0.03											
	*Listeria monocytogenes*	4.1								0.6	0.6–0.8										
*Y* *E* *A* *S* *T* *S*	*Candida albicans*	200	200	200	200					4.5–9	4.5–9								10	16	
	*Candida krusei*													10	0.3						
	*Candida parapsilosis*									4.5											
	*Trichosporon beigelii*									4.5											
	*Trichophyton rubrum*									9											
	*Malassezia furfur*									9											
	*Fusarium solani*										50										
	*Saccharomyces cerevisiae*	300	300	300	300															12	
	*Pichia anomala*													30	0.08						
	*Paecilomyces heliothis*																				12.5–25
	*Kluyveromyces thermotolerans*													3	0.3						
REFERENCES		[62,67]	[62]	[62]	[62]	[72]	[72]	[75]	[50]	[14,87,98,99,100]	[88]	[48]	[110]	[35,117]	[113]	[6]	[6,44]	[43]	[130]	[42]	[49]

**Table 4:** Amino acidic sequences hydrophobicity and net charge of BRICHOS-AMPs. In bold type, cysteine residues involved in disulfide bridges.

AMP Name	Amino Acid Sequence	Hydrophobicity	Net Charge At pH 7
Arenicin-1	RWCVYAYVRVRGVLVRYRRCW	42%	+6
Arenicin-2	RWCVYAYVRIRGVLVRYRRCW	42%	+6
Arenicin-3	GFCWYVCVYRNGVRVCYRRCN	28%	+4
Alvinellacin	RGCYTRCWKVGRNGRVCMRVCT	22%	+6
Nicomicin-1	GFWSSVWDGAKNVGTAIIKNAKVCVYAVCVSHK	45%	+3
Nicomicin-2	GFWSSVWDGAKNVGTAIIRNAKVCVYAVCVSHK	45%	+3
